# The long noncoding RNA TINCR promotes self-renewal of human liver cancer stem cells through autophagy activation

**DOI:** 10.1038/s41419-022-05424-1

**Published:** 2022-11-16

**Authors:** Jing Shi, Cao Guo, Yang Li, Junli Ma

**Affiliations:** 1 Affiliated Hospital of Qinghai University, Xining, 810001 Qinghai Province China; 2 Affiliated Hospital of Jining Medical University, Jining, 272029 Shandong China; 3 Institute of Medical Sciences, Xiangya Hospital, Central South University, Changsha, 410008 Hunan China

**Keywords:** Self-renewal, Prognostic markers

## Abstract

Hepatocellular carcinoma (HCC) is an extraordinarily heterogeneous tumor, which holds high recurrence and metastasis rates. Liver cancer stem cells (LCSCs) have been considered to be important influencing factors of these pathological properties, but the underlying mechanisms are poorly understood in HCC. Considerable evidences have shown that autophagy has an important role in cancer stemness. However, it is still unknown whether a long noncoding RNA (lncRNA) TINCR is involved in autophagy and self-renewal maintenance of HCC. In this study, TINCR was found to be highly expressed in HCC tissues and LCSCs. In vitro and in vivo assays for the first time showed that TINCR was required for LCSC self-renewal and tumorigenesis. Moreover, gene ontology analysis revealed the involvement of autophagy in the maintenance of TINCR-regulated stemness. Mechanically, TINCR was associated with polypyrimidine tract binding protein 1 (PTBP1) protein, which further promoted the transcription activity of autophagy related gene ATG5. In conclusion, we demonstrated that TINCR regulated LCSC self-renewal by autophagy activation through PTBP1/ATG5 regulatory pathway, offering a potential new target for HCC therapy.

## Introduction

Liver cancer, one of the most common human malignancies in the world, is the third leading cause of cancer-related deaths [1]. As the major histological subtype, hepatocellular carcinoma (HCC) accounts for approximately 90% of all cases. For all stages combined, HCC holds an increasing incidence and still low survival rate [2]. Despite advances in surgical and locoregional therapies, the 5-year survival rate of HCC patients remains unsatisfactory because of the high rates of recurrence and metastasis. Consequently, it is of much importance to explore the molecular mechanisms underlying HCC metastasis.

HCC is an extraordinarily heterogeneous tumor. Cancer stem cells (CSCs), subpopulations of cancer cells, have been considered to be important determining factors of intra-tumor heterogeneity and capable of tumor initiation and progression [3, 4]. These CSCs are responsible for tumor self-renewal, differentiation, and giving rise to a new tumor. Live CSCs (LCSCs) are also key factors in HCC carcinogenesis. Accumulating studies have shown that identifying targets that are critical for LCSCs function contributes to diagnosis and treatment of HCC [5, 6]. Biomarkers for LCSCs are critical players in regulating the properties of HCC, and considered to be the hopeful therapeutic targets [7]. However, the molecular mechanisms implicated in LCSC self-renewal remains elusive.

Autophagy is an adaptive catabolic process to maintain vital cellular functions during cell stress. It is found that autophagy is critical for the survival and stemness maintenance of CSCs and is an enhancer of CSC tumorigenesis [8]. CSCs are often characterized by high levels of autophagy that maintains CSCs pluripotency, regulates migration and invasion of CSCs and helps to escape immunosurveillance [9]. Moreover, autophagy induces CSCs resistance to chemoradiotherapy by promoting the dormant state of CSCs [10]. Consequently, identifying the regulatory factors and the molecular mechanisms of autophagy in CSCs is important to develop more effective antitumor strategies.

Long non-coding RNAs (lncRNAs) are classified as transcripts longer than 200 nucleotides, which have limited protein-coding potential, but can exert their functions at epigenetic, transcriptional, or post-transcriptional levels [11, 12]. In cancer, lncRNAs collaborate with protein complexes to act as regulators of transcription and mRNA stabilization [13, 14]. LncRNA has been reported to be implicated in a wide range of biological processes, including maintenance of stem cell properties and tumor progression [15, 16]. The functions and mechanisms of lncRNAs in CSCs are controversial, with most lncRNAs promoting the self-renewal of CSCs, while some lncRNAs exerting the opposite roles [17].

Tissue differentiation-inducing non-protein coding RNA (TINCR), located on chromosome 19p13.3, is association with a variety of cancers, including breast cancer, lung cancer, gastric cancer, and liver cancer [18, 19]. Notably, it is reported that TINCR exerts opposite roles in the pathogenesis of different human cancers [20]. The functions of TINCR on the biological properties of LCSCs are still unknown. In this study, the roles of TINCR in LCSCs are explored for the first time. It is found that TINCR is required to maintain self-renewal of LCSCs and tumor propagation. This finding might provide insights into the function and mechanisms underlying the regulation of HCC by TINCR.

## Materials and methods

### Patient samples and tissue specimens of HCC

A total of 54 pairs of fresh specimens of HCC and adjacent non-tumorous liver tissue (ANLT) were randomly acquired from HCC patients who underwent surgical resection at Affiliated Hospital of Jining Medical University from July 2020 to March 2021. All patients had not received any treatment prior to surgery. Matched fresh tissues were snap-frozen in liquid nitrogen for subsequent RNA extraction. The validation cohort (paraffin-embedded tissues, *n* = 89) were randomly collected from HCC patients receiving hepatic resection without other treatment prior to operation from January 2014 to December 2014. Paraffin-embedded HCC tissues were used to perform in situ hybridization (ISH) assay.

Patients were followed with regular surveillance *via* serum AFP measurement, CT scanning, and/or magnetic resonance imaging (MRI). Recurrence-free-survival (RFS) is calculated from the date of the surgery to the first recurrence. Overall survival (OS) was defined from assignment to death or the last observation. Patients that died not from recurrence or were still alive at the lat follow-up were censored. The study protocols were approved by the Ethics Committees of Affiliated Hospital of Jining Medical University.

### ISH

A hybridization probe specific for TINCR was designed and synthesized by Servicebio (Wuhan, China). The probe sequence for TINCR was as follows: 5′-FAM-AGTGCCTTCCAAAAGTGCCCTCTACCCCA-FAM-3′. Briefly, paraffin-embedded HCC tissue sections were deparaffinized, rehydrated and incubated with hybrid liquid overnight. After hybridization, the slides were incubated with biotin-labeled anti-digoxin, and the probe signal was visualized with diaminobenzidine solution. Then the fluorescence images were captured.

### Cell culture

HCCLM3 and MHCC97L cell lines were purchased from Procell, Wuhan, China and cultured in high glucose DMEM medium supplemented with 10% fetal bovine serum (GIBCO, Grand Island, NY).

### Flow cytometry and cell sorting

HCC cell lines and primary cells of HCC tissues were stained using PE-conjugated anti-human CD133 and FITC-conjugated anti-human CD13 antibodies according to the manufacturer’s instructions, and then detected using a FACS Calibur (BD Bioscience, San Jose, CA). For cell sorting, anti-human CD133 and anti-human CD13 antibodies were incubated with HCC cells, followed by being sorted with FACS Aria III (BD Bioscience).

### Sphere formation assay

Cells were cultured on ultra-low attachment culture dishes with serum-free medium. DMEM/F12 medium (Invitrogen) contained 2mM L-glutamine, 1% sodium pyruvate (Invitrogen), 20 ng/ml epithelial growth factor (EFG), 10 ng/ml fibroblast growth factor-2 (FGF2), and B27 (Invitrogen).

### Quantitative real-time polymerase chain reaction (qRT-PCR)

Total RNA was extracted from frozen tumor specimens or HCC cells using Trizol reagent (Invitrogen, Carlsbad, CA). SYBR Green fluorescent-based qRT-PCR was performed as previously described [21]. The primers are detailed in Supplementary Table S1. Relative RNA expression levels were calculated using the 2^−ΔΔCt^ method and normalized to the internal control.

### Cell fractionation assay

Cell fractionation assay was conducted according to the manufacturer’s protocol of cytoplasmic & nuclear RNA purification kit (Thorold, ON, Canada). Briefly, HCCLM3 and MHCC97L cells were lysed using lysis buffer J, and centrifuged to separate cell fractions. Then the supernatant was used for assessing the cytoplasmic RNA, and the pellet was used for nuclear RNA extraction. RNA expression were quantified by qRT-PCR assay and calculated by the 2^−ΔΔCt^ method. GAPDH and U6 were used as cytosolic and nuclear markers respectively.

### Western blotting

Treated cells were collected and lysed in RIPA lysis buffer (Beyotime, Shanghai, China) containing PMSF and phosphatase inhibitor. Total protein was collected, separated by SDS-PAGE gel, and transferred onto PVDF membrane (Millipore, Bedford, MA). After being blocked by 5% skim milk, the membrane was incubated with primary antibodies against LC3 (1:1000 dilution, #12741, CST), P62 (1:1000 dilution, ab109012, Abcam), ATG5 (1:1000 dilution; ab108327, Abcam), PTBP1 (1:5000 dilution, ab133734, Abcam), CD133 (1:1000 dilution, ab222782, Abcam), SOX2 (1:1000 dilution, ab171380, Abcam), TOM20 (1:1000 dilution, #42406 S, CST), Notch1 (1:1000 dilution, #3608S, CST), β-Catenin (1:1000 dilution, #8480 S, CST), SHH (1:1000 dilution, #2207S, CST), β-Actin (1:1000 dilution, #8457S, CST), and GAPDH (1:2000 dilution, 10494-1-AP, Proteintech), followed by HRP-conjugated secondary antibodies. The bands were visualized by luminescent imaging workstation (Tanon, Shanghai, China).

### Cell transfection

Three candidate shRNA sequences targeting TINCR were designed and synthesized by GeneChem (Shanghai, China), and the target sequences were listed in Supplementary Table S2. Negative control shRNA was purchased from GeneChem. Briefly, the cultured cells, with 50–70% density, were transfected with TINCR shRNA and shCtrl lentivirus with an optimal multiplicity of infection (MOI) of 20 TU/mL in the presence of polybrene (10 mg/mL, GeneChem, Shanghai, China). TINCR full-length cDNA was cloned into pcDNA3.1 vector and transfected cells. Expression of TINCR was confirmed by qRT-PCR.

### Transmission electron microscopy

The treated cells were fixed with gluteraldehydeand and postfixed with 2.5% osmium tetroxide. After being dehydrated, the samples were embedded in epon resin and cut into 80-nm sections. Imagines were examined by a transmission electron microscope (JEM-1230, Tokyo, Japan).

### Analysis of autophagic flux

To monitor the autophagic flux, the mRFP-GFP-LC3 adenovirus (HanBio Co. LTD, Shanghai, China) were transfected into LCSCs as previously described [21]. The images were captured using confocal fluorescence microscopy (Perkin-Elmer).

### RNA immunoprecipitation (RIP) assay

RIP was conducted according to the manufacturer’s instructions of a RIP^TM^ RNA-binding protein immunoprecipitation kit (Millipore, Cambridge, MA, USA). After being harvested and lysed in buffer, the cells were cultured with antibodies against polypyrimidine tract binding protein 1 (PTBP1) (1:1000 dilution, abcam) and immunoglobulin G overnight at 4 °C. Then streptavidin-coated magnetic beads were added, and the purified RNA were subjected to qRT-PCR analysis.

### Xenograft growth in nude mice

The mice (4–6 weeks old, male, BALB/c) were randomly divided (*n* = 5–6). Different dilutions of control and transfected cells were implanted into the flank regions of mice respectively. Tumor growth was monitored every three days and tumor volume was calculated as: Volume (mm^3^) = (length × width^2^) × 0.5. The experiment was approved by the Animal Use Committee of Affiliated Hospital of Jining Medical University. The mice were treated humanely during the whole study period.

### Statistical analysis

The statistical analysis was performed using GraphPad Prism 5.0/9.0 (Graphpad Software, La Jolla, CA) and SPSS 17 (SPSS, Inc, Chicago, IL). Chi-Squared test was performed to assess the correlation between TINCR expression and clinicopathologic features. Kaplan-Meier method was used for survival curves and Cox regression model was established to analyze survival-related factors. Student’s *t*-test and one-way ANOVA test were applied to assess differences between different groups. Statistical significance was set at *p* < 0.05.

## Results

### TINCR was highly expressed in HCC tissues and LCSCs

**Fig. 1:**
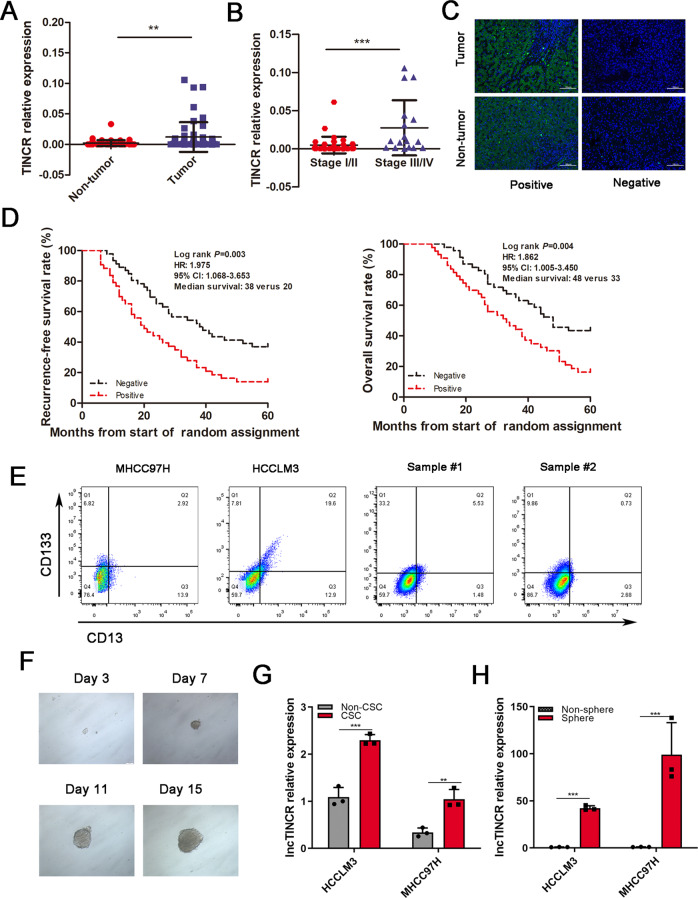
TINCR was highly expressed in liver tissues and CSCs. **A** TINCR expression levels in HCC tissues were assessed by qRT-PCR assay. TINCR expression in tumor tissues were higher than the matched normal tissues (*p* < 0.01). **B** The patients with III/IV stage exhibited higher TINCR expression in tumor tissues than those with stage I/II. **C** TINCR expression was determined by ISH assay. Representative images of TINCR in HCC and adjacent tissue are shown. Scale bar, 100 μm. **D** RFS and OS of HCC patients in validation cohort. Patients with positive TINCR expression had shorter RFS (*p* = 0.003) and OS (*p* = 0.004). **E** CD133^+^CD13^+^ subpopulations were sorted by flow cytometry from HCCLM3, MHCC97H HCC cell lines, and primary tumor cells. **F** Typical microscope images of spheres in HCCLM3 cells. Scale bar, 100 mm. **G** TINCR was detected in LCSCs and non-LCSCs by qRT-PCR assay. **H** TINCR expression levels were examined in oncosphere and non-oncosphere cells. Data are shown as means ± SD and represent at least three independent experiments. **p < 0.01, ***p < 0.001.

We evaluated the expression of TINCR in 54 paired frozen HCC specimens in the training cohort by qRT-PCR. The findings demonstrate that TINCR expression was significantly elevated in HCC tissues than that in ANLT (*p* < 0.01, Fig. 1A). HCC patients at stage III/IV exhibited higher TINCR expression levels in HCC tissues than those at stages I/II (*p* < 0.001, Fig. 1B). To verify the expression characteristics of TINCR in HCC patients, ISH was performed in 89 cases in the validation cohort (Fig. 1C). Consistent with the training cohort, TINCR expression in HCC was higher compared with the corresponding adjacent tissues (*p* < 0.05), and associated with TNM stage and presence of venous invasion (Table 1).

**Table 1:** Relationship between TINCR expression and the clinical pathological indexes of HCC.

Clinical pathological indexes		Validation Cohort (89 cases)		
		Low	High	*P*
Age (years)	≤54	19	26	0.055
	> 54	27	17	
Gender	Male	39	34	0.335
	Female	7	9	
Albumin	≤35 g/L	42	35	0.164
	> 35 g/L	4	8	
Child-Pugh classification	A	32	32	0.393
	B	14	11	
AFP (ng/ml)	≤20	38	30	0.074
	> 20	8	13	
Liver cirrhosis	Absent	29	33	0.120
	Present	17	10	
Tumor size (cm)	≤5	25	22	0.465
	> 5	21	21	
Tumor number	Single	37	34	0.541
	Multiple	9	9	
Venous invasion	Absent	42	31	0.018^*^
	Present	4	12	
Tumor differentiation	I-II	31	33	0.229
	III-IV	15	10	
TNM stage	I-II	42	32	0.032^*^
	III	4	11	

*HCC* Hepatocellular carcinoma, *SD* Standard deviation, *HBsAg* Hepatitis B surface antigen, *AFP* A-fetoprotein. ^*^Significant difference (*p* < 0.05) is shown in bold.

A multivariable Cox regression analysis revealed that TNM stage (HR = 3.182, *p* = 0.013; HR = 3.473, *p* = 0.010), venous invasion (HR = 2.306, *p* = 0.019; HR = 2.500, *p* = 0.009) and TINCR (HR = 1.975, *p* = 0.030; HR = 1.862, *p* = 0.0348) were independent prognostic factors of RFS and OS in validation cohort, respectively (Table 2). Kaplan-Meier survival analysis showed that the median RFS of HCC patients with positive TINCR expression was significantly lower than those subjects with negative TINCR expression (38 ± 7.35 months vs. 20 ± 3.93 months, *p* = 0.003). Median OS in these two groups were 48 ± 5.31 months and 33 ± 5.90 months respectively, with statistical significance (*p* = 0.004; Fig. 1D). These data showed that TINCR might be a poor prognostic factor and involved in HCC progression.

**Table 2:** Multivariate analysis by a Cox proportional hazards regression model.

Variable	PFS		OS	
	HR (95% CI)	*p*	HR (95% CI)	*p*
Age (≤54 y vs. >54 y)	1.006(0.974-1.039)	0.404	0.994(0.568-1.739)	0.982
Gender (male *vs*. female)	0.739(0.363-1.504)	0.708	0.683(0.327-1.425)	0.309
Albumin (≤35 g/L vs. >35 g/L)	0.684(0.314-1.493)	0.341	0.499 (0.213-1.167)	0.109
AFP ( ≤ 400ug/L vs. >400 ug/L)	0.957(0.490-1.870)	0.898	0.929 (0.469-1.841)	0.833
Child-Pugh classification (A vs. B)	1.146(0.634-2.074)	0.652	1.178(0.646-2.149)	0.593
Liver cirrhosis (absence vs. presence)	1.375(0.721-2.623)	0.333	1.170(0.624-2.196)	0.624
Tumor differentiation (I / II vs. III/ IV)	0.847(0.457-1.569)	0.597	0.947(0.509-1.759)	0.863
Tumor size (≤5 cm vs. >5 cm)	1.362(0.795-2.333)	0.260	1.352(0.771-2.371)	0.293
Tumor number (single vs. multiple)	1.137(0.463-2.794)	0.779	1.132(0.450-2.847)	0.793
TNM stage (I–II vs. III–IV)	3.182(1.276-7.939)	0.013^*^	3.473(1.339-9.010)	0.010^*^
Venous invasion (absence vs. presence)	2.306(1.145-4.647)	0.019^*^	2.500(1.253-4.987)	0.009^*^
TINCR expression (negative vs. positive)	1.975(1.068-3.653)	0.030^*^	1.862(1.005-3.450)	0.048^*^

*CI* Confidence interval, *HR* Hazard ratio, ^*^Significant difference (*p* < 0.05) is shown in bold.

Next, TINCR in HCC cells was also explored. CD133 and CD13 have been widely used as LCSCs surface markers [5, 22], so CD13^+^CD133^+^ subpopulations were sorted from HCC cell lines and primary samples, followed by flow cytometry analysis (Supplementary Fig. S1, Fig. 1E). Then the sorted LCSCs were performed to conduct sphere-formation assays to investigate the expression of TINCR in LCSCs (Fig. 1F). The results showed that TINCR was highly expressed in LCSCs, and more higher in oncosphere cells derived from HCCLM3 and MHCC97L cells (Fig. 1G, H). These data indicated that TINCR was highly expressed in HCC tumor tissues and LCSCs and might play important roles in HCC development.

### TINCR maintained self-renewal of LCSCs

**Fig. 2:**
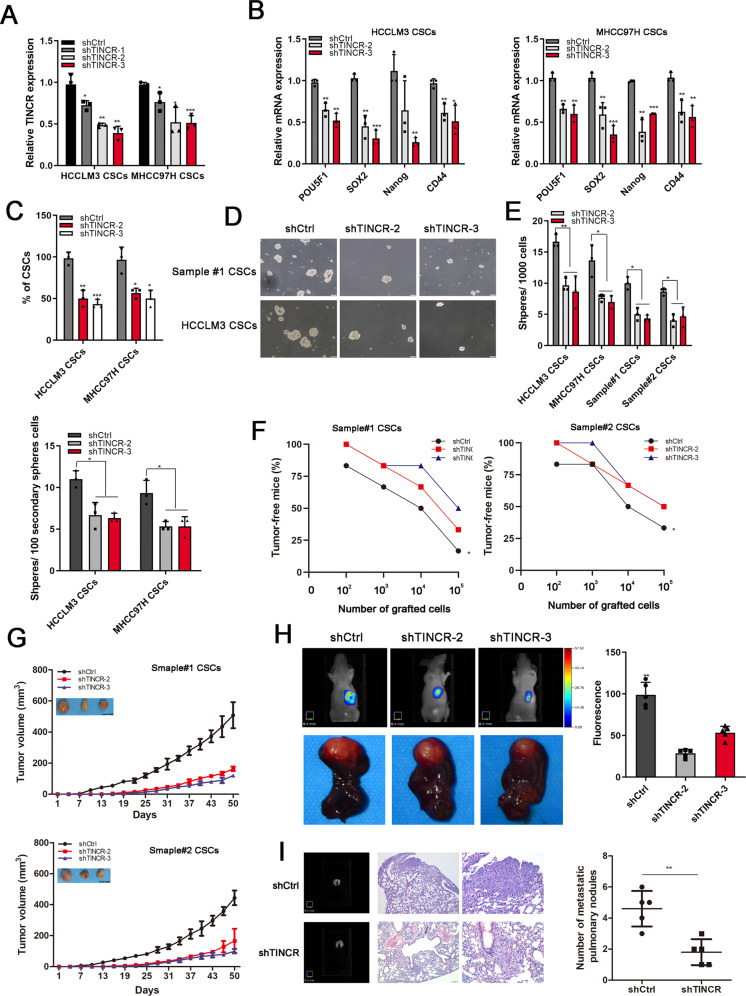
TINCR maintained self-renewal of LCSCs. **A** TINCR expression was silenced in LCSCs by three independent shRNAs and assessed by qRT-PCR. **B** Transcription factors (POU5F1, SOX2, Nanog, and CD44) were analyzed by qRT-PCR in TINCR-depleted cells. **C** CD13^+^CD133^+^ subpopulations (CSCs) were reduced when TINCR was silenced. **D** The typical morphological features of spheres after knockdown of TINCR. Scale bar, 100 mm. **E** Quantification of the total number of primary and secondary spheres derived from single LCSCs after TINCR knockdown, compared with the control cells. **F** Different dilutions of TINCR depleted cells were subcutaneously implanted into BALB/c mice. *n* = 6 for each group. **G** Tumor growth curves of subcutaneous implantation models of HCC. **H** The representative liver orthotopic tumor images scanned by fluorescence molecular tomography imaging system. **I** Representative lung metastasis pictures of fluorescent imaging and HE staining (Scale bar, 200 μm & 50 μm). Data are shown as means ± SD. *p < 0.05, **p < 0.01, ***p < 0.001.

To explore the role of TINCR in LCSCs, lentivirus-mediated short hairpins RNAs (shRNAs) were used to infected LCSCs, with shRNA-2 and shRNA-3 achieving more effective knockdown efficiency (Fig. 2A). When TINCR was knocked down, the expression of transcription factors POU5F1, SOX2, Nanog, and surface marker CD44 were reduced (Fig. 2B). Moreover, the amount of stemness marker SOX2 was explored by immunofluorescence assay and the result showed a significant increase in LCSCs, but not in non-CSCs. Furthermore, TINCR knockdown remarkably reduced SOX2 signals in LCSCs (Supplementary Fig. S2). We next found that TINCR depletion could remarkably impaired the production of CD13^+^CD133^+^ subpopulation (CSCs) (Fig. 2C). Additionally, we observed the number of primary sphere and secondary sphere formation were reduced after TINCR knockdown (Fig. 2D, E).

In vivo, different dilutions of stable knockdown of TINCR and shCtrl HCC primary cells were used to investigate the role of TINCR in tumor-initiating formation. As shown in Fig. 2F, silencing of TINCR needed more grafted cells inducing tumors and exhibited a weaker tumorigenic capacity, compared with the control groups (Supplementary Fig. S3). During the monitoring of tumor growth, TINCR depletion resulted in significantly decreased tumor growth and smaller final tumor size (Fig. 2G). Furtherly, liver orthotopic tumors model was established to show that TINCR knockdown exhibited decreased tumor size and lower fluorescence intensities (Fig. 2H). Fluorescent imaging analysis and hematoxylin-eosin staining (HE) images confirmed that knockdown of TINCR significantly attenuated pulmonary metastasis (Fig. 2I). Overall, TINCR silencing reduced the tumorigenic capacity of LCSCs.

### TINCR overexpression enhanced tumorigenic capacity of LCSCs

**Fig. 3:**
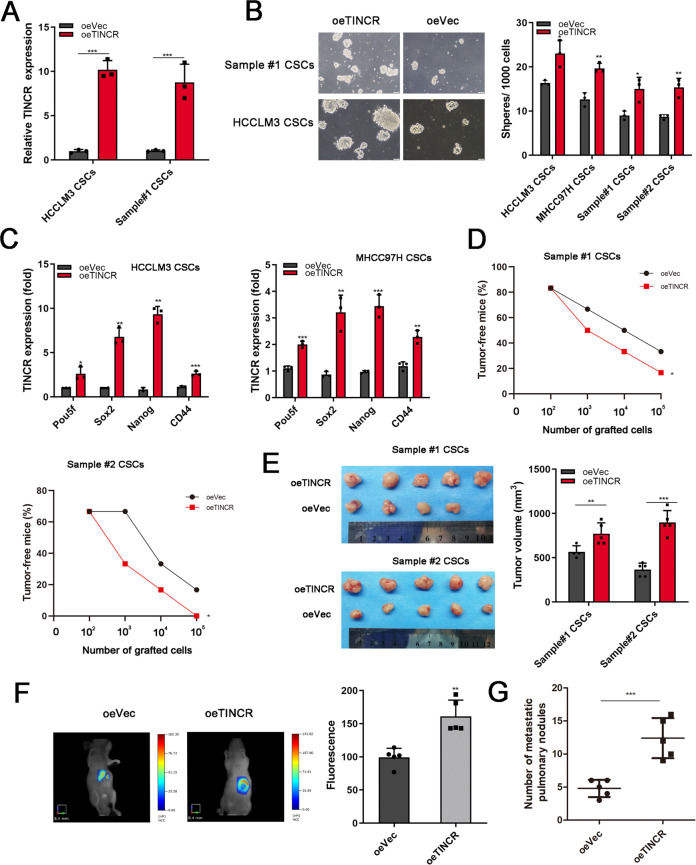
Overexpressed TINCR enhanced tumorigenic capacity of LCSCs. **A** TINCR stably overexpressed HCC primary tumor cells and cell lines were established. (B) TINCR overexpression promoted sphere formation of HCC cell lines and primary cells. Scale bar, 100 mm. **C** Expression of transcription factors were elevated in TINCR-overexpressing HCC cells. **D** TINCR overexpressed primary tumor cells were diluted and subcutaneously implanted into BALB/c mice. *n* = 6 for each group. (E) After subcutaneous implantation, the average tumor volumes in each groups were calculated. **F** Fluorescence signals of liver orthotopic tumor from TINCR-overexpressing and empty vector groups were captured. **G** TINCR-overexpressing promoted lung metastasis rate. Data are shown as means ± SD. *p < 0.05, **p < 0.01, ***p < 0.001.

We next established TINCR stably overexpressing HCC primary tumor cells and cell lines (Fig. 3A). Overexpressed TINCR promoted oncosphere formation and expression of CSCs related genes POU5F1, SOX2, Nanog, and CD44 (Fig. 3B, C). Notably, enhanced SOX2 signals were observed in LCSCs with TINCR overexpression by immunofluorescence assay (Supplementary Fig. S2). Additionally, TINCR enhanced tumor-initiating capacity (Fig. 3D) and xenograft tumor volumes (sample 1: 0.57 ± 0.03 cm^3^ vs. 0.77 ± 0.05 cm^3^, *p* < 0.01; sample 2: 0.37 ± 0.03 cm^3^ vs. 0.90 ± 0.06 cm^3^, *p* < 0.001, Fig. 3E). Consequently, TINCR overexpression dramatically augmented fluorescence intensities in xenograft tumors (Fig. 3F). Moreover, pulmonary metastasis rate was found to be increased when TINCR was upregulated (Fig. 3G). All together, these data indicated that TINCR was critical for self-renewal and tumorigenic capacity of LCSCs.

### Autophagy involved in TINCR regulation of LCSCs

**Fig. 4:**
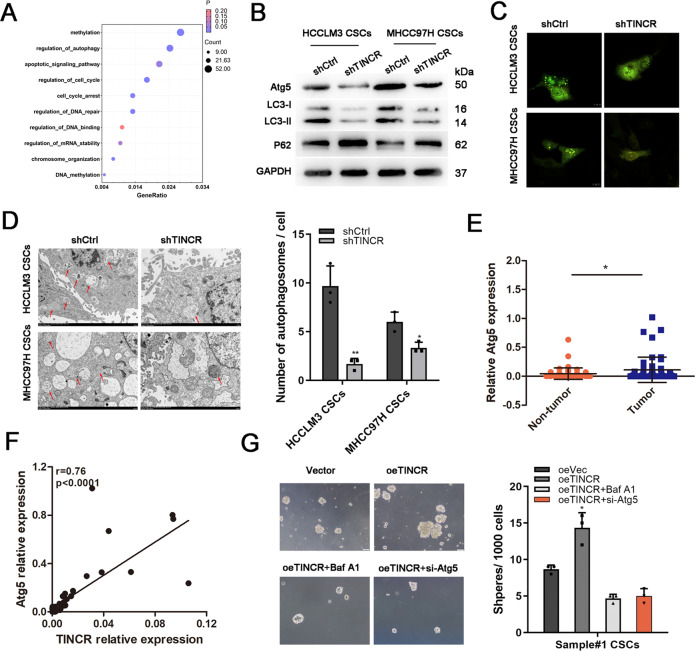
TINCR regulated ATG5-mediated autophagy. **A** GO pathway analysis showed the involvement of autophagy in TINCR regulation. **B** After TINCR depletion, the level of autophagy-related proteins were detected by western blotting. **C** LC3 fluorescence intensity was assessed by confocal microscope. Scale bar: 10 μm. **D** Representative images of autophagosome detected by TEM. **E** ATG5 expression in HCC tissues was evaluated by qRT-PCR. **F** ATG5 expression was positively correlated with TINCR expression (*r* = 0.76, *p* < 0.0001). **G** When LCSCs were treated with Baf-A1 (75 nM) or ATG5 siRNA, sphere formation was attenuated both in these two groups. Data are shown as means ± SD. *p < 0.05.

Through RNA sequencing and gene ontology analysis (GSE 215894), we hypothesized that autophagy might be involved in the function regulation of TINCR (Fig. 4A). Importantly, ATG5, a biomarker for autophagy, was one of the genes screened for variability. Furtherly, ATG5 was identified to be targeted by TINCR (Fig. 4B). In order to explore the regulation of TINCR on autophagy, western blot was carried out to demonstrate the down-regulated levels of LC3 and up-regulated levels of p62 when TINCR was knocked down (Fig. 4B). The immunofluorescence assay showed that mRFP-GFP-LC3 puncta distributions, representing fluorescence intensity of the LC3 protein, were remarkably attenuated in TINCR silencing cells (Fig. 4C). Transmission electron microscopy (TEM) was further performed to observe that autophagic density was significantly reduced in LCSCs from HCCLM3 and MHCC97H cells after TINCR knockdown (Fig. 4D).

To further explore the clinical implications of autophagy related gene ATG5 in HCC, we detected the expression of ATG5 by qRT-PCR assay, and found that it was upregulated in HCC tissues, compared with ANLT (*P* < 0.05, Fig. 4E). There was an positive correlation between TINCR and ATG5 expressions in these specimens (*R* = 0.76, *p* < 0.0001, Fig. 4F). Knockdown of ATG5 in LCSCs resulted in a decrease in the size and number of mammospheres formed, suggesting that ATG5 was critical for maintaining the proliferation of LCSCs (Fig. 4G). The link between autophagy and the maintenance of LCSCs was further confirmed by lysosomal inhibitor Baf-A1, an inhibitors of autophagy. As the result, self-renewal capacity promoted by overexpressed TINCR was attenuated by Baf-A1 (Fig. 4G). Overall, these data suggested that ATG5 involved in spheres formation and we believed that autophagy might play an important role in TINCR-regulated self-renewing of LCSCs.

As a representative of selective autophagy, mitophagy was explored also. Recently, mitophagy has been directly implicated in maintaining the stem cell state [23, 24]. Here, we explored whether similar to autophagy, mitophagy was responsible for TINCR-regulated self-renewal in LCSCs. We treated LCSCs with Mdivi-1, a mitophagy inhibitor, and carbonyl cyanide m-chlorophenyl hydrazone (CCCP), a mitophagy inducer [23]. As shown in Supplementary Fig. S4A, Mdivi-1 decreased the levels of mitochondrion-associated LC3-II, whereas CCCP had the opposite effects. But Mdivi-1 and CCCP both failed to change the expression levels of stemness-related proteins CD133 and SOX2 (Supplementary Fig. S4B). Besides, there was no significant difference in self-renewal ability (Supplementary Fig. S4C). Our data suggested that non-selective autophagy, but not mitophagy, likely contributed to LCSCs stemness.

### TINCR interacted with PTBP1 to stabilize ATG5 mRNA

**Fig. 5:**
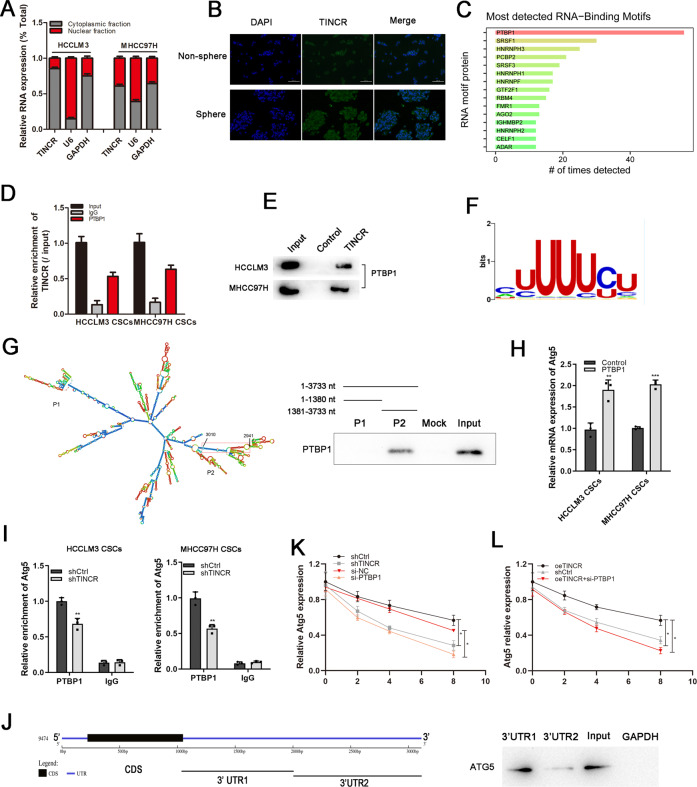
TINCR stabilized ATG5 mRNA by interacting with PTBP1. **A**, **B** Cellular fractionation and RNA-ISH assay were conducted to found cytoplasmic localization of TINCR. Scale bar, 100 mm. **C** catRAPID omics predicted the protein-RNA interactions. **D**, **E** RNA pull-down showed that PTBP1 interacted with TINCR. **F** RBPsuite (http://www.csbio.sjtu.edu.cn/bioinf/RBPsuite/) predicted the sequence motifs of PTBP1 binding sites. **G** RNAalifold (http://rna.tbi.univie.ac.at/cgi-bin/RNAWebSuite/RNAalifold.cgi) predicted the secondary structure of TINCR. The PTBP1 binding stem-loop structures in TINCR was indicated by red border. Pull-down assay and western blot were then performed to identify regions required for TINCR and PTBP1 interaction. **H** Relative ATG5 mRNA was detected by qRT-PCR when PTBP1 was overexpressed. **I** RIP assay was performed to detect the interaction between ATG5 and PTBP1 after TINCR knockdown. **J** Schematic description of the ATG5 mRNA 5′UTR, CDS, and 3′UTR. The interaction between PTBP1 and ATG5 mRNA 3′UTR was examined by pull-down analysis. **K** ATG5 mRNA stability was assessed in LCSCs with silencing TINCR or PTBP1 after being treated with actinomycin D. **L** ATG5 mRNA stability was evaluated in cells with overexpressed TINCR or combined with PTPB1 knockdown. Data are shown as means ± SD. *p < 0.05, **p < 0.01, ***p < 0.001.

We used cellular fractionation assays and RNA fluorescence in situ hybridization (RNA-ISH) to find that TINCR was mainly localized in the cytoplasm of HCC tumor cells (Fig. 5A, B). Based on an online catRAPID evaluation, the binding proteins of TINCR was predicted, and PTBP1 was the most detected RNA binding protein (RBP) (Fig. 5C). RNA pull-down and RIP assay were further utilized to validate the specific interaction between TINCR and PTBP1 (Fig. 5D, E). Sequence analysis by RBPsuite predicted a sequence motif of the RBP binding site for PTBP1 (Fig. 5F), which was located in the 2941-3010nt region of TINCR to form a stem-loop structure. To determine the precise interacting part, we designed the oligonucleotide probes based on the predicted secondary structure of PTBP1 and observed that P2 fragment (1381-3733 nt) was the main region responsible for binding TINCR (Fig. 5G). Then, we sought to prove that TINCR interacted with ATG5 by binding with PTBP1. It was suggested that when PTBP1 was upregulated, ATG5 expression was observably increased (Fig. 5H). Furthermore, we analyzed whether TINCR affected the interaction between PTBP1 and ATG5, and finally found that silencing of TINCR attenuated the interaction between PTBP1 and ATG5 (Fig. 5I). Since RBPs usually interact with the 3′UTR of target mRNA and affect to its stability, we investigated the interaction between PTBP1 and ATG5 3′UTR (3′UTR1 and 2). As shown in Fig. 5J, the result confirmed that PTBP1 could bind to the 3′UTR1 mRNA transcript of ATG5. Subsequently, ATG5 mRNA stability was assessed in LCSCs treated with Actinomycin D. The results showed that silencing TINCR or PTBP1 all could lead to an increased degradation of ATG5 mRNA (Fig. 5K). By contrast, overexpression of TINCR could enhance ATG5 stability, which was attenuated after co-transfection with silencing PTBP1 (Fig. 5K). So the results indicated that TINCR regulated ATG5 mRNA stability and increased the expression levels of ATG5 by interacting with PTBP1.

### TINCR promoted self-renewal of LCSCs by regulating PTBP1-mediated autophagy

**Fig. 6:**
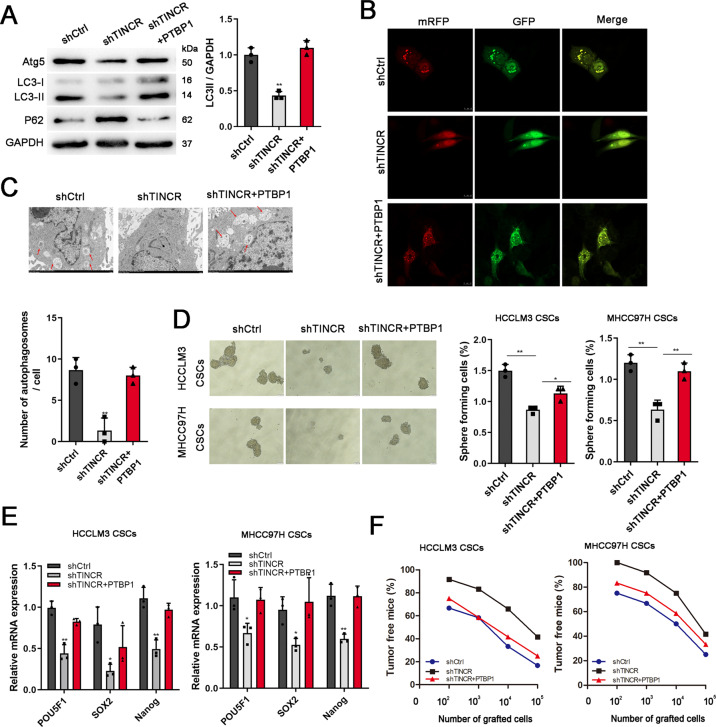
TINCR promoted self-renewal of LCSCs by regulating PTBP1-mediated autophagy. PTBP1 overexpressed vector and shRNA targeting TINCR co-transfected LCSCs. Involvement of PTBP1 was evaluated by western blotting (**A**), confocal microscope (**B**, scale bar: 10 μm), and TEM assay (**C**). **D** PTBP1 overexpression restored the oncosphere formation ability reduced by TINCR depletion. **E** Stem factors (POU5F1, SOX2, Nanog) were detected by qRT-PCR in the treated LCSCs. **F** The treated LCSCs derived from HCCLM3 and MHCC97H were diluted and subcutaneously implanted into BALB/c nude mice. Data are shown as means ± SD. *p < 0.05, **p < 0.01.

To demonstrate the role of PTBP1 in TINCR regulation, we co-transfected PTBP1 overexpressed vector and shRNA targeting TINCR into LCSCs, and found that PTBP1 could rescue autophagy inhibition caused by shTINCR (Fig. 6A–C). Notably, PTBP1 recovered the oncosphere formation ability reduced by TINCR depletion (Fig. 6D). PTBP1 overexpression dramatically increased expression of pluripotent transcription factors influenced by TINCR knockdown (Fig. 6E). Additionally, in vivo study showed that PTBP1 promoted tumor-initiating capacity reduced by TINCR depletion (Fig. 6F). All together, our data show that TINCR activates the transcription of ATG5 by interacting with PTBP1, leading to LCSC self-renewal and tumor propagation.

## Discussion

CSCs harbor the stem properties of self-renewal, differentiation and the ability to form tumors [25, 26]. CSCs are often identified from the cell surface markers. A number of surface markers of LCSC subpopulations have been explored, such as EpCAM, CD133, CD44, and so on [27, 28]. However, none of these markers is exclusively expressed in LCSCs, and definite LCSC markers are still controversial [29]. In this study, CD133 and CD13 in subpopulation of cells from HCC cell lines and primary cells were found to be significantly elevated and used to identify LCSCs. It is found that LCSCs possess the capability of circulation within the body, which significantly promotes distant metastasis and homing ability, compared with other tumor cell types [5, 30]. Consistently, our findings reveal that LCSCs promote growth of primary cancer cells and metastasis of transplanted secondary tumors, leading to HCC progression. CSCs display specific features, and contribute to tumor onset, recurrence and metastasis, so especially targeting CSCs may be one promising therapeutic strategy against cancer. CSCs-targeted therapeutic strategies include targeting cell surface markers, signaling pathways, and differentiation therapy of CSCs [31].

An increasing number of studies have demonstrated the critical modulating role of lncRNAs in tumor stemness phenotype [32, 33]. Here, we identified an lncRNA TINCR, which was highly expressed in HCC tissues and LCSCs. It is reported that TINCR performing functions in human malignancies is tissues-specific [16, 34]. In HCC, we demonstrated that the abnormally upexpressed TINCR play a vital role in priming the self-renewal of LCSCs. LncRNAs exert their functions *via* diverse mechanisms, including cotranscriptional regulation and modulation of gene expression. Downstream molecular mechanisms of TINCR in LCSCs were investigated through GO analysis, and it was found that autophagy was involved in TINCR-regulated stemness. Autophagy is an intracellular process that maintains cellular homeostasis. Recently, autophagy has been related to CSCs and cancer metastasis by promoting stem cell phenotype [35–37]. The regulation of autophagy involves several conserved autophagy-related genes. ATG5 has been previously found to be involved in the initiation, formation, extension and closure stages of autophagy [38, 39]. In our study, blockade of autophagy by pharmacological approaches or ATG5 inhibitors reduced the self-renewal ability, suggesting the role of autophagy in LCSCs maintenance. However, the detailed mechanism by which autophagy is connected to CSCs is still under investigation. The maintenance of CSCs self-renewal is a very complicated biological process, in which the Notch, Hedgehog (HH), and Wnt pathways are important signals [6, 40]. In our study, when ATG5 was knocked down, Sonic Hedgehog expression was decreased, but β-catenin and Notch1 expression did not change significantly (Supplementary Fig. S5). It suggested that autophagy might regulate LCSC activity by modulating Hedgehog signalings, which need to be further confirmed.

Cytoplasmic localization of TINCR indicates the downstream pathways mainly at the post-transcriptional level, in which there are two regulatory patterns: regulating mRNA stability by interacting with RBPs and sequestering miRNAs [41, 42]. To determine the underlying mechanism of TINCR-regulated ATG5, we identified a RBP PTBP1 that could interact with TINCR. PTBP1 belongs to the heterogeneous nuclear ribonucleoproteins (hnRNPs) family and regulates pre-RNA processing, especially alternative splicing [43]. As a multifunctional RBP and splicing factor, PTBP1 regulates posttranscriptional gene expression and involves in mRNA splicing, stability, and localization. PTBP1 has been detected to be an oncogene and associated with a range of cancers, including hepatocellular carcinoma [44, 45]. Wang H et al. found that PTBP1 involved in HCC progression by facilitating SETD7/LZTFL1 mRNA destabilization [45]. Here, we discovered that LCSCs self-renewal ability was influenced by TINCR binding to PTBP1 to alter the stability of transcripts related to autophagy.

In conclusion, TINCR can promote LCSC self-renewal and tumor progression by autophagy activation through PTBP1/ATG5 regulatory pathway. The precise regulatory model and critical roles of tissue context and tumor microenvironment need to be fully elucidated.

## Supplementary information

Supplementary Fig. Legends

Supplementary Fig. S1

Supplementary Fig. S2

Supplementary Fig. S3

Supplementary Fig. S4

Supplementary Fig. S5

Supplementary Table S1

Supplementary Table S2

Original Data File 3

Checklist

## Data Availability

The datasets used and/or analysed during the current study are available from the corresponding author on reasonable request.
