# Supplementary material for: The long noncoding RNA TINCR promotes self-renewal of human liver cancer stem cells through autophagy activation
Source: Cell Death Dis. 2022 Nov 16;13(11):961. doi: 10.1038/s41419-022-05424-1 (PMC9668904; doi:10.1038/s41419-022-05424-1)
Supplement: Supplementary file 1 — Supplementary Fig. Legends [file 41419_2022_5424_MOESM1_ESM.docx]

**Figure S1**

**Sphere form ability of HCC cells.** CD13^+^CD133^+^ subpopulations were analyzed and sorted from HCC cell lines by flow cytometry.

**Figure S2**

**TINCR promotes stemness marker SOX2 expression.** When TINCR was knocked down (A) or overexpressed (B), immunofluorescence assay was conducted to the expression of TINCR and SOX2.

**Figure S3**

**In vivo limiting dilution experiments display the dumorigenic role of TINCR.** Tumor pictures for each group are provided when TINCR was knocked down (A) or overexpressed (B).

**Figure S4**

**Effects of mitophagy on hepatic CSCs.** LCSCs were treated with Mdivi-1 or CCCP for 24 hours. (A) Cells were subjected to subcellular fractionation for the isolation of mitochondria and immunoblot analysis. (B) Immunoblot analysis of CD133 and SOX2. (C) Sphere-forming abilities were analyzed after being treated by Mdivi-1 or CCCP.

**Figure S5**

**The Sonic hedgehog signaling pathway is involved in regulation of LCSC activity by autophagy.** Cells with ATG5 knockdown were lysed for immunoblot analysis.
