# Supplementary figures and images for: The long noncoding RNA TINCR promotes self-renewal of human liver cancer stem cells through autophagy activation
Source: Cell Death Dis. 2022 Nov 16;13(11):961. doi: 10.1038/s41419-022-05424-1 (PMC9668904; doi:10.1038/s41419-022-05424-1)

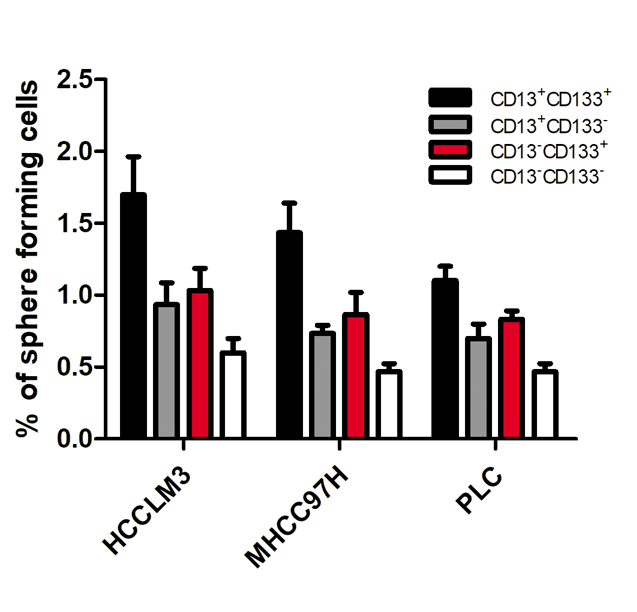

Supplement: Supplementary file 2 — Supplementary Fig. S1 [file 41419_2022_5424_MOESM2_ESM.tif]

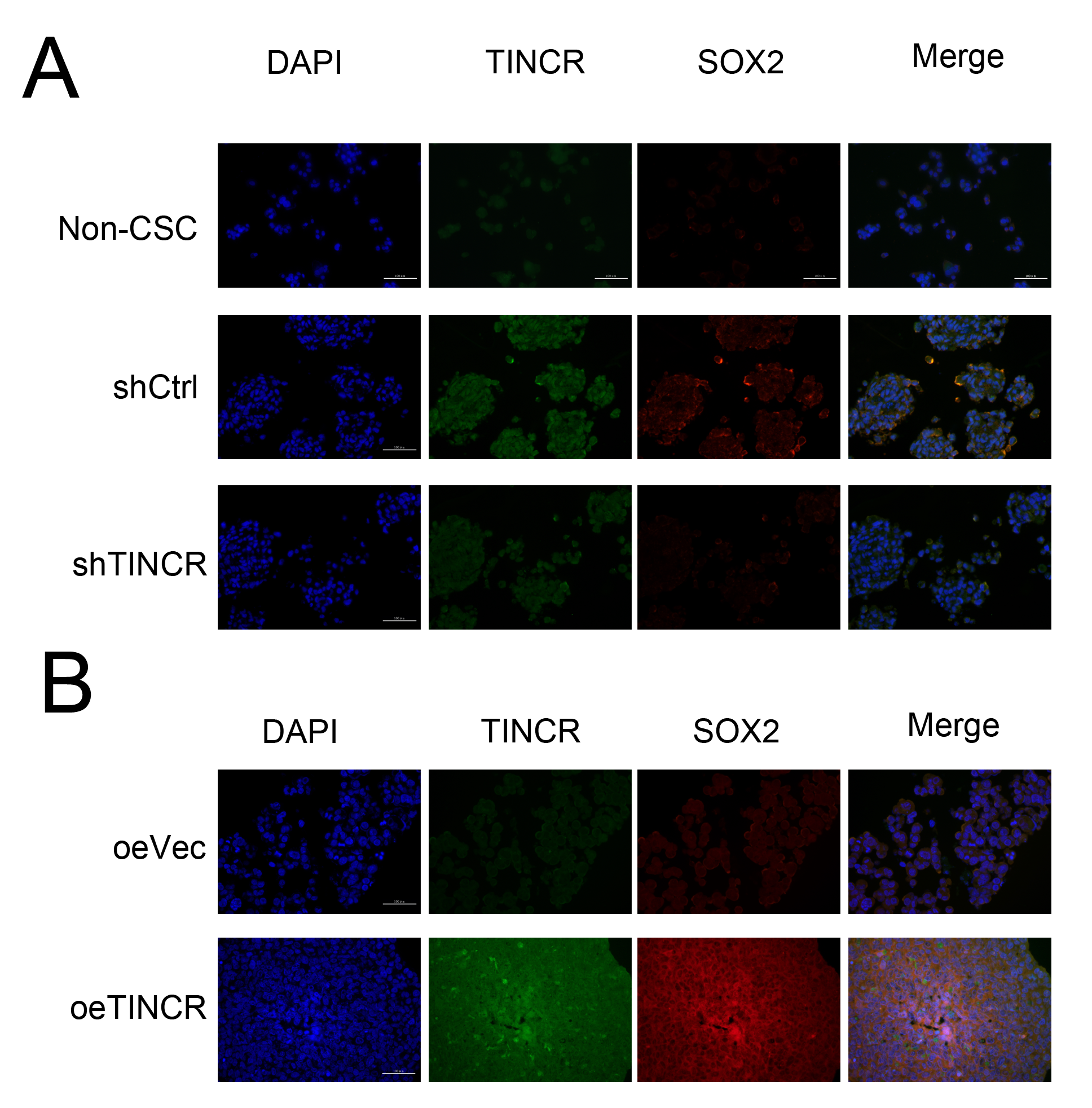

Supplement: Supplementary file 3 — Supplementary Fig. S2 [file 41419_2022_5424_MOESM3_ESM.tif]

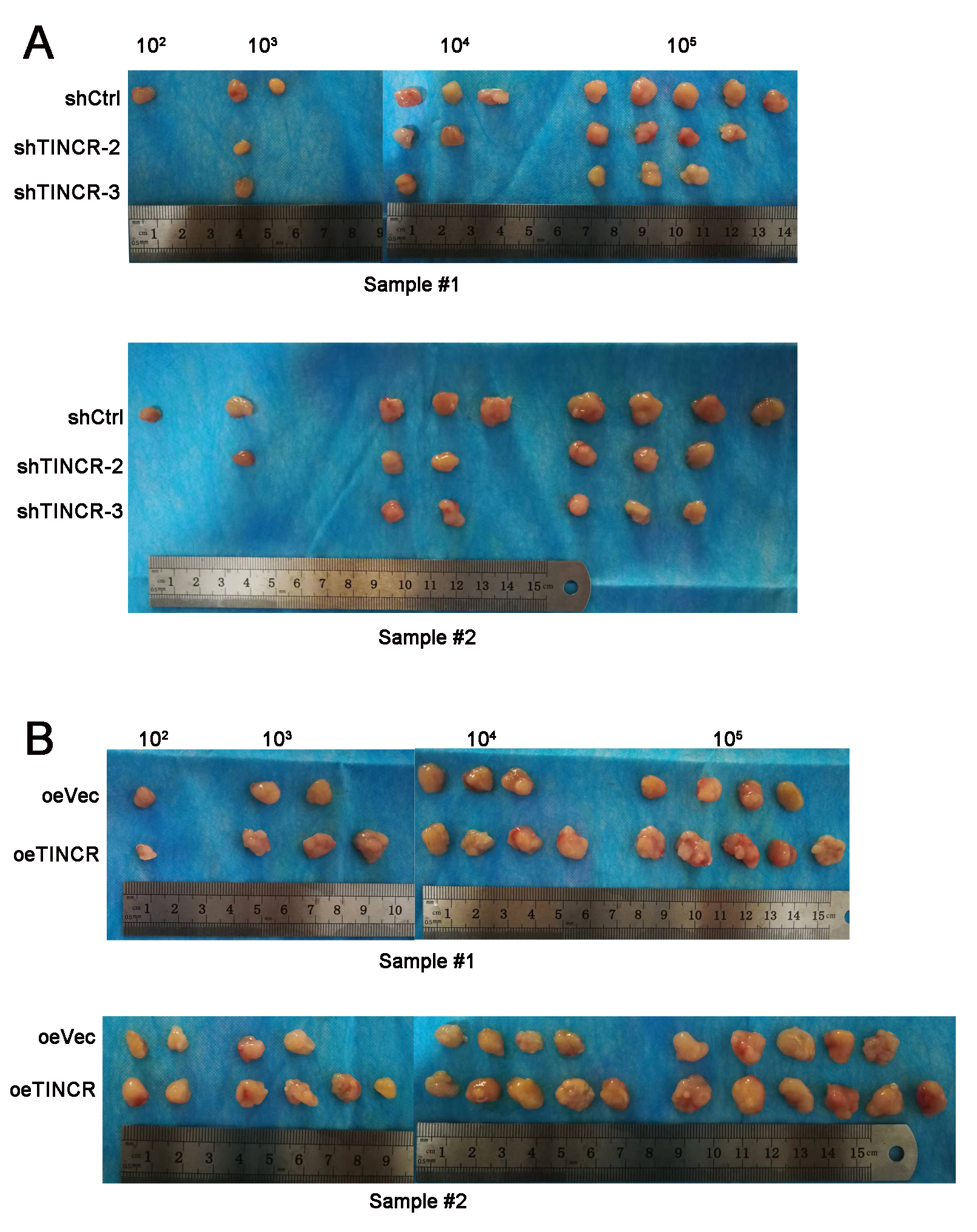

Supplement: Supplementary file 4 — Supplementary Fig. S3 [file 41419_2022_5424_MOESM4_ESM.tif]

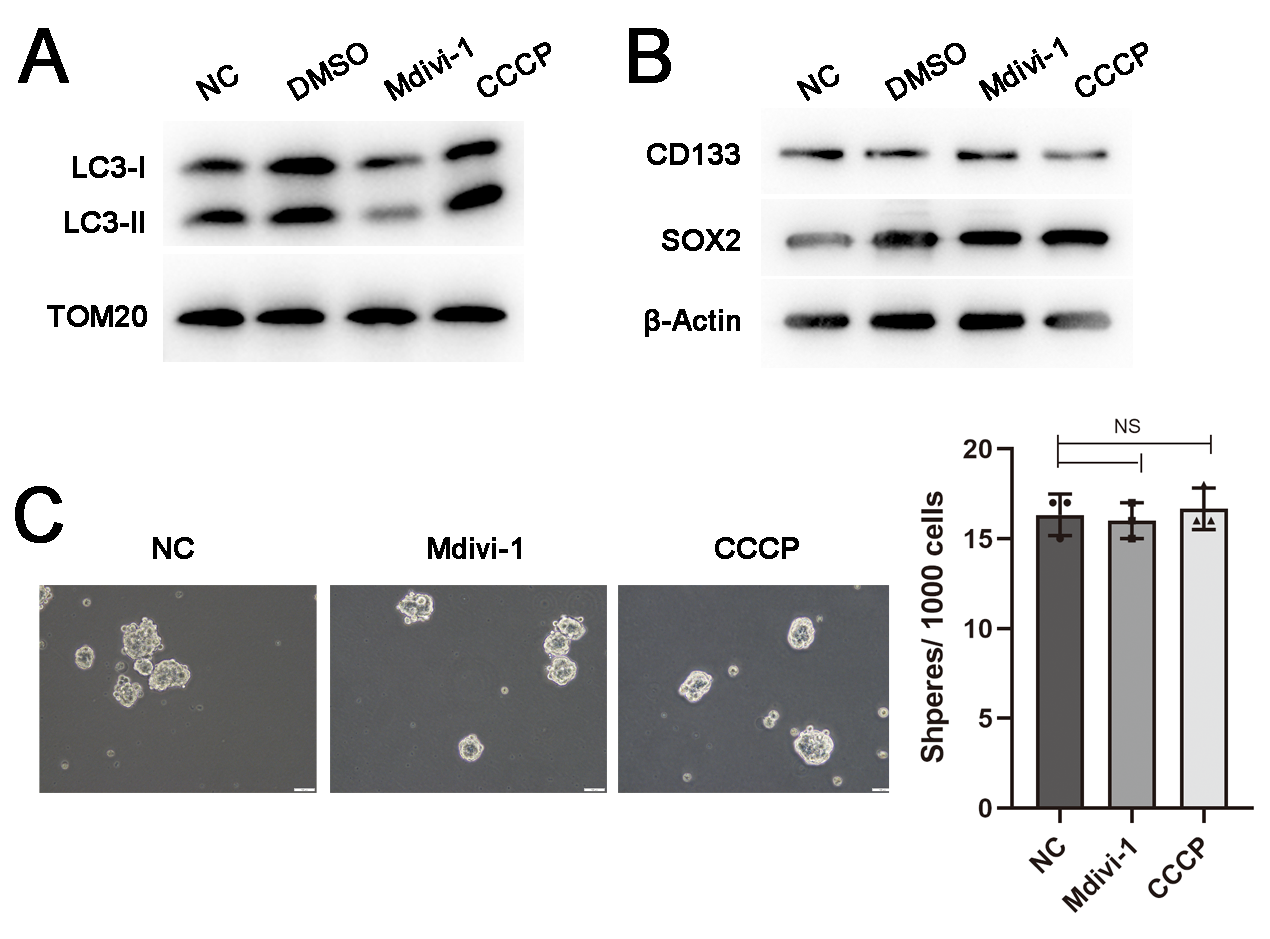

Supplement: Supplementary file 5 — Supplementary Fig. S4 [file 41419_2022_5424_MOESM5_ESM.tif]

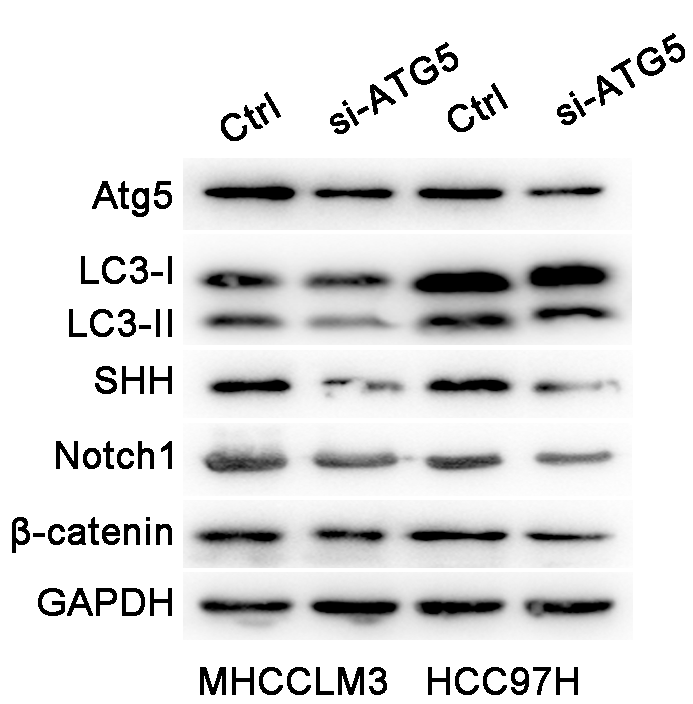

Supplement: Supplementary file 6 — Supplementary Fig. S5 [file 41419_2022_5424_MOESM6_ESM.tif]
