# Supplementary material for: The long noncoding RNA TINCR promotes self-renewal of human liver cancer stem cells through autophagy activation
Source: Cell Death Dis. 2022 Nov 16;13(11):961. doi: 10.1038/s41419-022-05424-1 (PMC9668904; doi:10.1038/s41419-022-05424-1)
Supplement: Supplementary file 7 — Supplementary Table S1 [file 41419_2022_5424_MOESM7_ESM.docx]

Supplementary Table S1. Oligonucleotide Sequences of Quantitative Real-Time RT-PCR Primers.

| **Gene symbol** | **sequence** |
| --- | --- |
| TINCR | F:5’-CTTGGTGCCTAACCCAGGACTTTG -3’ |
|  | R:5’-GCAGAACAGACAGCAGCAGACTC -3’ |
| Pou5f1 | F:5’- GTGGTCCGAGTGTGGTTCTGTAAC -3’ |
|  | R:5’- CCCAGCAGCCTCAAAATCCTCTC -3’ |
| Sox2 | F:5’- CAGCATGTCCTACTCGCAGCAG -3’ |
|  | R:5’- CTGGAGTGGGAGGAAGAGGTAACC -3’ |
| Nanog | F:5’- GATGCAAGAACTCTCCAACATC -3’ |
|  | R:5’- CTGGTGGTAGGAAGAGTAAAGG -3’ |
| CD44 | F:5’-TCTGAATCAGATGGACACTCAC-3’ |
|  | R:5’-CATTGCCACTGTTGATCACTAG-3’ |
| Atg5 | F:5’-AAGCAACTCTGGATGGGATT-3’ |
|  | R:5’-GCAGCCACAGGACGA AAC-3’ |
| U6 | F:5’-CTCGCTTCGGCAGCACA-3’ |
|  | R:5’-AACGCTTCACGAATTTGCGT-3’ |
| GAPDH | F:5’-CGGAGTCAACGGATTTGGTCGTAT-3’ |
|  | R:5’-AGCCTTCTCCATGGTGGTGAAGAC -3’ |
