# Supplementary material for: The long noncoding RNA TINCR promotes self-renewal of human liver cancer stem cells through autophagy activation
Source: Cell Death Dis. 2022 Nov 16;13(11):961. doi: 10.1038/s41419-022-05424-1 (PMC9668904; doi:10.1038/s41419-022-05424-1)
Supplement: Supplementary file 8 — Supplementary Table S2 [file 41419_2022_5424_MOESM8_ESM.docx]

**Supplementary Table S2：Target Seq of TINCR**

| **NO.** | **Sequence** | **Titer** |
| --- | --- | --- |
| TINCR RNAi-1 | caGCTTGGAACTAGATACAGA | 1E+9 |
| TINCR RNAi-2 | TAGCTTCAATACCTGCTACTT | 1E+9 |
| TINCR RNAi-3 | CAGCGCTGGCATGTTCTGAAA | 1E+9 |
