# Supplementary material for: The long noncoding RNA TINCR promotes self-renewal of human liver cancer stem cells through autophagy activation
Source: Cell Death Dis. 2022 Nov 16;13(11):961. doi: 10.1038/s41419-022-05424-1 (PMC9668904; doi:10.1038/s41419-022-05424-1)

Original western blots of Figure 4B

Atg5

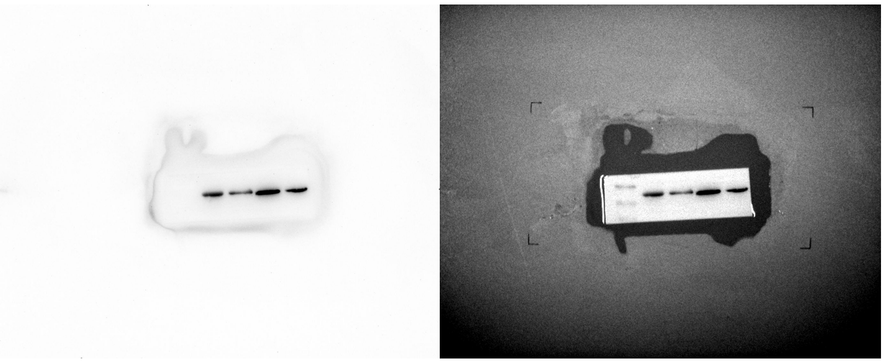

LC3

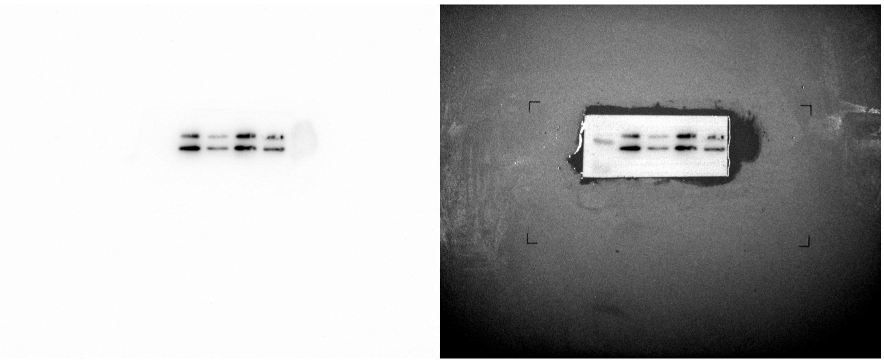

P62

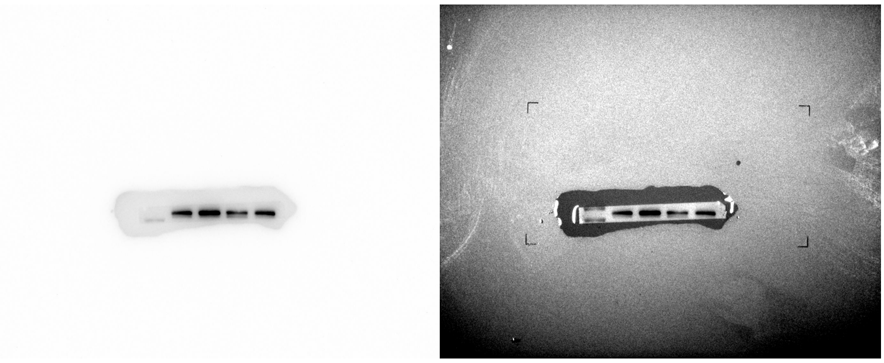

Gapdh

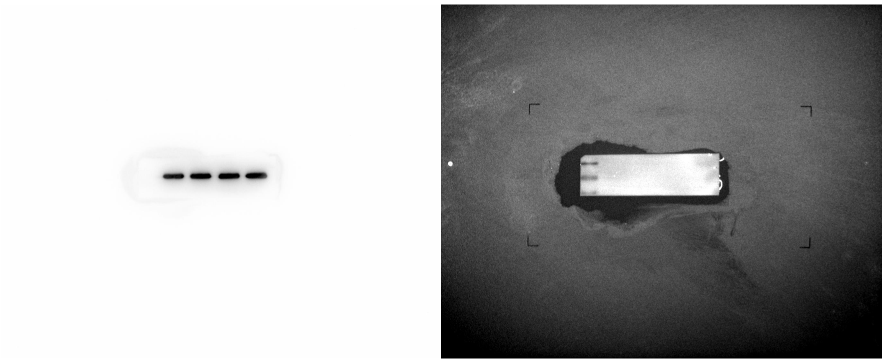

Original western blots of Figure 6A

Four lanes of the western blots were shCtrl, shTINCR, shTINCR+PTBP1, and shTINCR+Atg5.Our main purpose in figure 6 is to explore the role of PTBP1 in TINCR-mediated autophagy, so we only chose the first three lanes. The original western blots of figure 6A were as follows.

Atg5

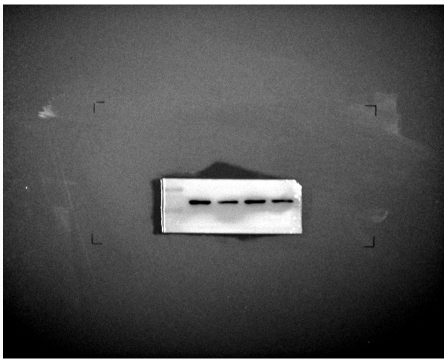

LC3

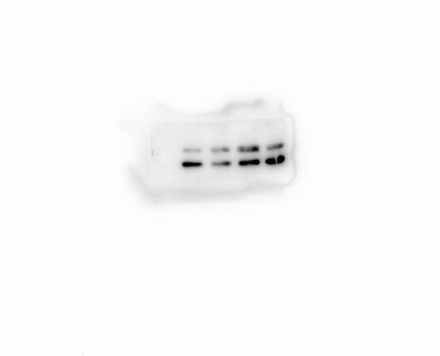

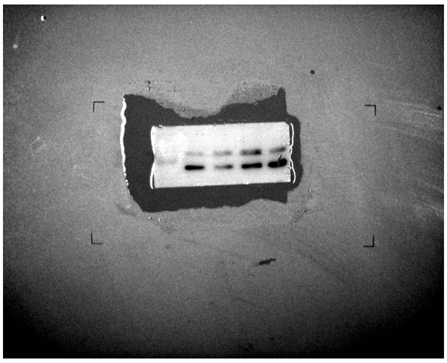

P62

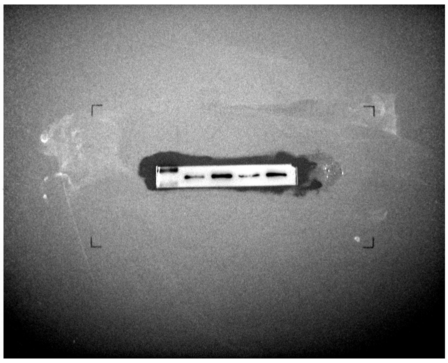

GAPDH

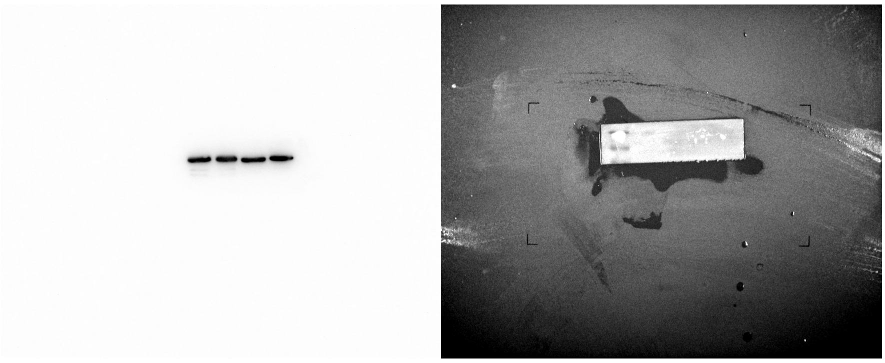

Original western blots of supplentary figure S4A

LC3

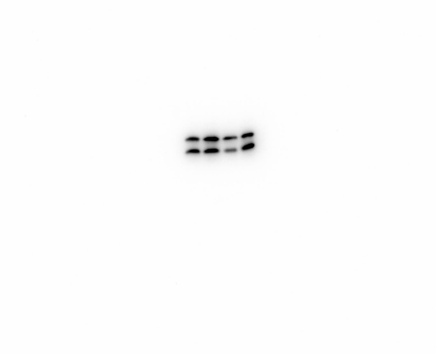
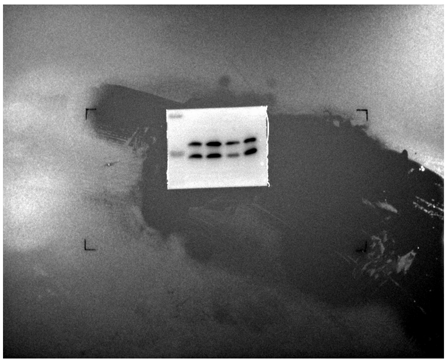

TOM20

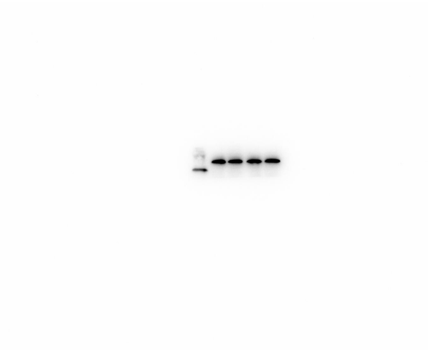
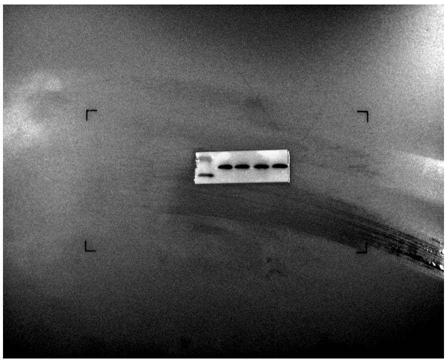

Original western blots of supplentary figure S4B

CD133

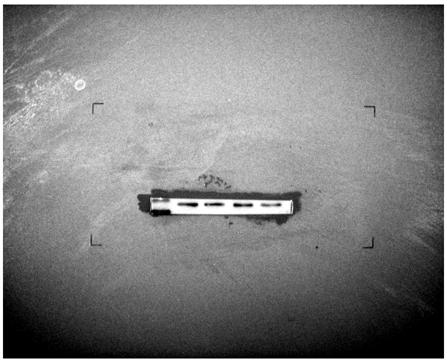

SOX2

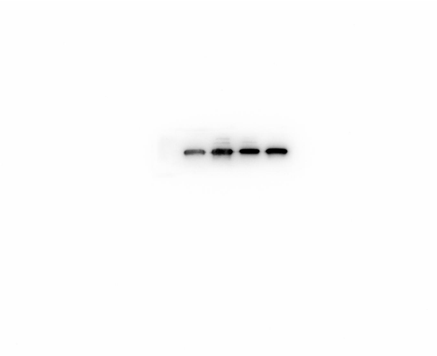

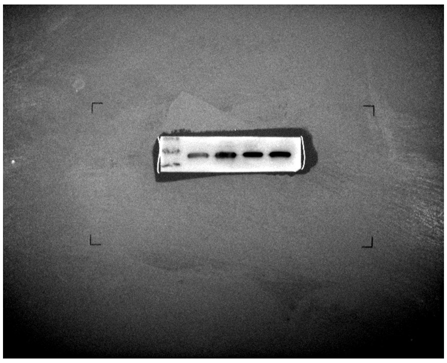

β-actin

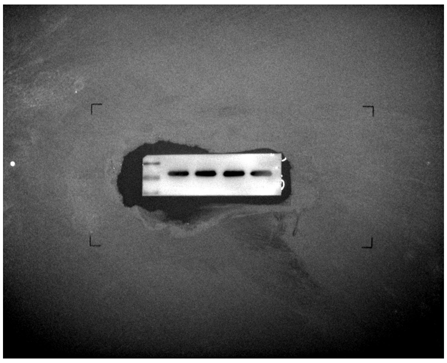

Original western blots of supplentary figure S5

ATG5

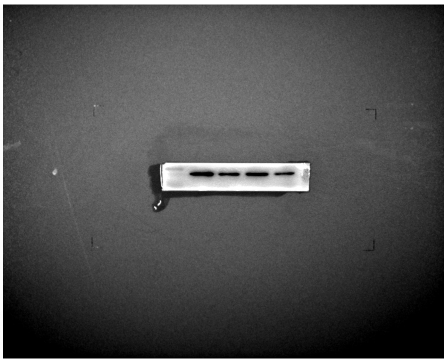

LC3

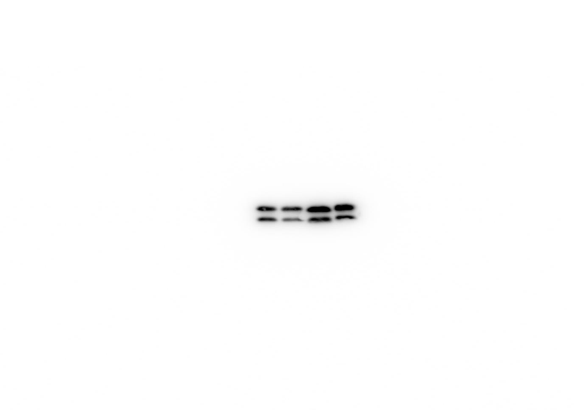

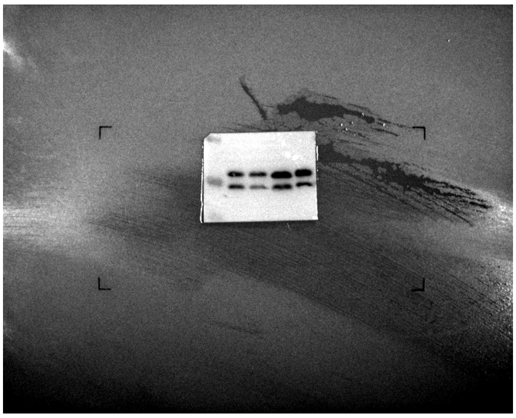

SHH

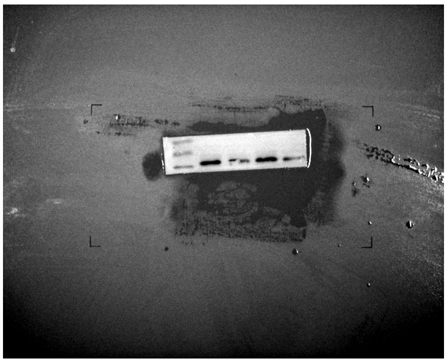

Notch1

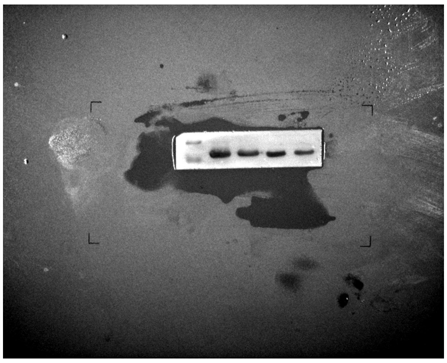

β-Catenin

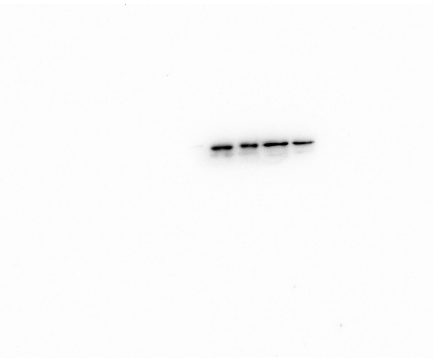

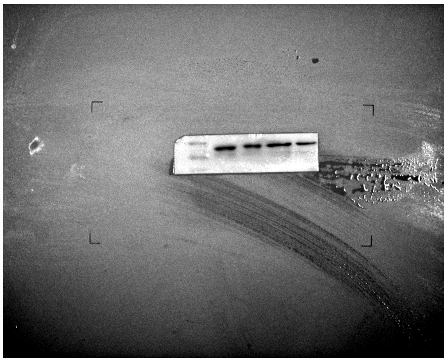

GAPDH

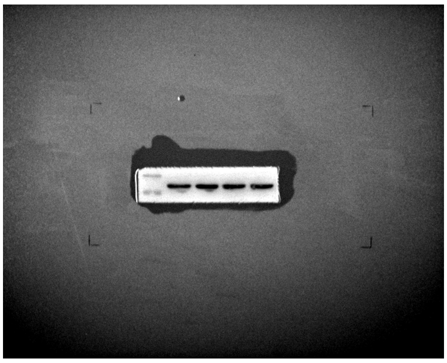

Supplement: Supplementary file 9 — Original Data File 3 [file 41419_2022_5424_MOESM9_ESM.pdf]
